# Potential effect of human chorionic gonadotropin‐induced extracellular vesicle RNAs on mouse ovarian granulosa cells

**DOI:** 10.1002/2211-5463.70367

**Published:** 2026-11-12

**Authors:** Natchanok Puangjit, Nobuhiko Takahashi, Yuki Maruyama, Shota Mayama, Katsueki Ogiwara, Chika Fujimori, Atsushi P. Kimura

**Affiliations:** ^1^ Graduate School of Life Science Hokkaido University Sapporo Japan; ^2^ Division of Internal Medicine, Department of Human Biology and Pathophysiology, School of Dentistry Health Sciences University of Hokkaido Ishikari‐Tobetsu Japan; ^3^ Department of Biological Sciences, Faculty of Science Hokkaido University Sapporo Japan

**Keywords:** extracellular vesicle, granulosa cell, Lhcgr, luteinizing hormone, mouse, RNA‐sequencing

## Abstract

Gonadotropins regulate granulosa‐cell function during ovarian follicular development, and extracellular vesicles (EVs) are potential mediators of intercellular communication within the follicle. However, the regulation of EV RNA cargo following gonadotropin stimulation remains incompletely understood. Here, we investigated human chorionic gonadotropin (hCG)‐responsive EV RNA profiles using three experimental models: two *Lhcgr*‐expressing clones of a mouse granulosa‐cell tumor‐derived cell line and mouse ovaries. The cell models were treated with hCG, and immature mice were primed with serotropin, followed by hCG. EVs were isolated from these cells and ovaries and characterized by transmission electron microscopy and western blot analysis. Total RNAs from these EVs were analyzed by electrophoresis, suggesting global changes in their profiles with hCG. RNA‐sequencing identified thousands of potentially differentially expressed genes, including 111 commonly upregulated and 122 commonly downregulated transcripts among all three experimental models. The 111 upregulated transcripts were further narrowed down to 27 candidates based on the abundance, and five protein‐coding transcripts (*Ccdc200*, *Gm10337*, *Gm614*, *Adad2*, and *Spata45*) were selected for further functional validation. Interestingly, EVs carrying the overexpressed *Ccdc200*, *Gm10337*, and *Gm614* mRNA significantly increased the number of ovarian cells during 3‐day culture. These findings indicate that hCG modulates EV RNA cargo and suggest that EV‐mediated RNAs cooperatively function as a regulatory layer of hCG signaling for intercellular communication, fine‐tuning granulosa‐cell proliferation or survival during follicular development.

## Graphical abstract

To mimic the LH action in the ovary, granulosa‐cell and mouse models were stimulated by hCG, which changed RNA profiles within extracellular vesicles (EVs), and potentially hCG‐responsive EV RNAs increased the number of recipient ovarian cells. EV‐associated RNAs are potential intermediaries of hCG signaling. Icons used in the illustrations were obtained from BioRender.

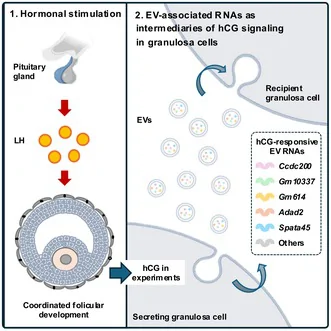

**Abbreviations**
DEG: differentially expressed gene
EV: extracellular vesicle
FSH: follicle‐stimulating hormone
hCG: human chorionic gonadotropin
LH: luteinizing hormone
Lhcgr: luteinizing hormone/choriogonadotropin receptor
miRNA: microRNA
ORF: open reading frame
PAGE: polyacrylamide gel electrophoresis
PBS: phosphate‐buffered saline
PCR: polymerase chain reaction
PVDF: polyvinylidene difluoride
qRT‐PCR: quantitative reverse transcription polymerase chain reaction
RNA‐seq: RNA‐sequencing
ROI: region of interest
RT‐PCR: reverse transcription polymerase chain reaction
SDS: sodium dodecyl sulfate
SEM: standard error of the mean
TBS‐T: Tris Buffered Saline with Tween 20
TEM: transmission electron microscopy
TPM: transcripts per million

Ovarian follicular development is a highly coordinated process involving proliferation and differentiation of granulosa cells, oocyte maturation, and dynamic interactions among multiple follicular cell types [1, 2, 3, 4]. The process is regulated by endocrine signals as well as local intra‐ovarian communication networks that control follicle growth and ovulation [5, 6]. Among the hormonal regulators, follicle‐stimulating hormone (FSH) is a major regulator of granulosa‐cell proliferation and differentiation during follicular development, and luteinizing hormone (LH) plays a key role in triggering the final stages of follicular maturation and ovulation [6, 7]. LH mediates downstream signaling cascades essential for ovulation through activation of a G protein‐coupled receptor, the luteinizing hormone/choriogonadotropin receptor (LHCGR) [8, 9, 10]. The *Lhcgr* gene is constitutively expressed in theca cells throughout the estrous cycle, and granulosa cells acquire *Lhcgr* expression in response to FSH during later stages of follicular development [11, 12, 13]. This results in further promotion of granulosa‐cell proliferation by FSH, and granulosa cells become ready for responding to the preovulatory LH surge [6, 7, 14, 15]. After LH surge, granulosa‐cell proliferation stops, and several events, such as cumulus expansion, meiosis resumption, and tissue remodeling are induced [16, 17]. At the molecular level, binding of LH to LHCGR activates cAMP‐dependent signaling pathways that induce rapid transcription of immediate‐early genes, such as *Egr1* and *Fos*, which also contribute to ovulatory processes [18, 19]. While intracellular signaling events downstream of LHCGR have been extensively studied, less is known about whether LH stimulation modulates extracellular communication mechanisms within the ovarian microenvironment.

Human chorionic gonadotropin (hCG) is commonly used experimentally to mimic the LH surge because it activates LHCGR and induces ovulatory responses. The reason why many researchers use hCG instead of LH is that hCG is more stable and cost‐effective. However, LH and hCG should not be considered functionally equivalent aside from stability and price. Studies comparing the two ligands have demonstrated differences in their potency, kinetics, and activation of intracellular pathways, including cAMP, ERK1/2, AKT, and β‐arrestin‐dependent signaling [20, 21, 22]. More recent work further supports ligand‐specific differences in LHCGR trafficking and postreceptor signaling [23]. Nonetheless, conducting research with hCG is of great significance because many researchers have collected a bunch of data with hCG.

One class of regulators that has recently attracted considerable attention in intercellular communication is extracellular vesicles (EVs). EVs, including exosomes, are membrane‐bound vesicles approximately 30–150 nm in diameter released by most cell types and are capable of transferring bioactive molecules, such as proteins, lipids, and RNAs to recipient cells [24, 25, 26, 27]. Increasing evidence suggests that EVs play important roles in ovarian physiology; especially EVs in follicular fluid are thought to mediate communication among granulosa cells, theca cells, and oocytes within the follicular environment [28, 29, 30]. Most studies that investigated molecular mechanisms underlying such communication have focused on the profiling of small noncoding RNAs, particularly microRNAs (miRNAs), suggesting their possible roles in the regulation of pathways involved in follicular development and oocyte maturation before and after LH surge or hCG treatment [31, 32, 33, 34, 35, 36]. Moreover, some studies proved the presence of functional mRNAs within EVs from follicular fluid before LH surge [37, 38]. In contrast, the presence and potential functions of protein‐coding RNAs and long noncoding RNAs within ovarian EVs after LH surge or hCG treatment remain less well characterized.

Given that hormonal stimulation has been shown to influence EV secretion and cargo composition in several biological systems [39, 40, 41], hCG or LH signaling can alter the mRNA cargo of ovarian EVs, but this issue has not been systematically investigated. To address this, here we established granulosa cell‐derived culture models that overexpressed *Lhcgr* and used them to analyze EV RNA composition, together with hormone‐treated mouse ovaries. Using EV RNA sequencing, we identified a subset of EV RNAs that were potentially altered by hCG treatment across culture models and mouse ovary. Based on findings that LH contributes to the regulation of granulosa cells [6, 7, 14, 15, 16, 17] and that ovarian EVs affect granulosa‐cell proliferation [30, 42, 43], we tested potential effects of selected protein‐coding EV RNA candidates on the number of cultured cells. Our findings provide insight into potential hCG‐dependent changes in EV RNA composition and suggest a possible role for EV‐mediated RNA dynamics in ovarian‐cell communication.

## Materials and methods

### Animals and hormone treatment

The Crl:CD1 (ICR) mice were kept on a 12‐h light and 12‐h dark cycle at 25 °C, with access to food and water available at all times. The experimental procedures for this study were approved by the Institutional Animal Care and Use Committee at Hokkaido University (approval no.: 19‐0043 and 24‐0048) and followed the ARRIVE 2.0 guidelines, including the essential 10. Mice were euthanized by cervical dislocation by well‐skilled researchers in accordance with the guidelines when necessary.

To activate LHCGR, we used hCG (ASKA Animal Health Co., Ltd., Tokyo, Japan) for both cell culture models and mice. Female immature ICR mice (4–5 weeks old) were randomly assigned to hCG‐treated and control groups. All mice received an intraperitoneal injection of 5 IU serotropin (ASKA Animal Health Co., Ltd.), followed 48 h later by an intraperitoneal injection of 5 IU hCG or phosphate‐buffered saline (PBS). Ovaries were collected 2 h postinjection, washed, and homogenized in cold PBS, and immediately processed for EV isolation and EV RNA extraction.

### Cell culture and hormone treatment

OV3121 cells that were derived from mouse ovarian granulosa‐cell tumor (JCRB1026; JCRB Cell Bank, Osaka, Japan) [44] were cultured in 10‐cm dishes in DMEM (Fujifilm Wako, Osaka, Japan) supplemented with 10% fetal bovine serum (Thermo Fisher Scientific, Waltham, MA, USA) and 100 U·mL^−1^ penicillin, 100 μg·mL^−1^ streptomycin, and 292 μg·mL^−1^ L‐glutamine (Nacalai tesque, Kyoto, Japan) at 37 °C in a humidified atmosphere of 5% CO_2_. OV3121‐LHR cells were established as described in the next section and cultured in the same way as OV3121 cells. These cultured cells were periodically authenticated in the past 3 years, based on morphological inspection, proliferation patterns, and expression of key genes. All experiments were performed with mycoplasma‐free cells.

When cells reached > 90% confluence, cultures were treated with 1 IU·mL^−1^ hCG or PBS as a control in serum‐free Opti‐MEM (Thermo Fisher Scientific) for 2 or 24 h. Conditioned media and cells were collected separately for EV isolation and cellular and EV RNA extraction.

### Establishment of OV3121 cell clones that stably transfected with the *Lhcgr* gene

The open reading frame (ORF) of mouse *Lhcgr* gene was obtained by reverse transcription polymerase chain reaction (RT‐PCR) with ovary cDNA using a primer pair shown in Table 1. The PCR product was inserted into a pBluescript II KS(+) vector at the EcoRV site and confirmed by DNA sequencing. The ORF sequence was cut out from the plasmid by digestion with EcoRI and SalI, blunted by T4 DNA polymerase, and ligated into pPBhCMV*1‐cHA‐pA which was digested with BglII and blunted.

**Table 1:** Primers used in this study.

Description	Product size (bp)	Forward	Reverse
[RT‐PCR]			
Lhcgr ORF	1672 bp	5′‐ATGGGGCGGCGGGTCCCG‐3′	5′‐GTTACTGAATTAACACTCTTGG‐3′
mLHRex11	722 bp	5′‐ATAAACTGACGGTGCCCCGC‐3′	5′‐TGCCTTCGTGAACACTGCG‐3′
Ccdc200	554 bp	5′‐ATGGGTAGTGCCTACCACTG‐3′	5′‐TCATCAAGTCTTGGAGTCCG‐3′
Gm10337	165 bp	5′‐TACCAGATGTCAACCTTCTC‐3′	5′‐CAGCATGCTAATCACATACG‐3′
Gm614	748 bp	5′‐GTCTGCCTAGTCTGGGCCTC‐3′	5′‐TGCTGCTCTCACTTGGTCTT‐3′
Adad2	1686 bp	5′‐ATGGCGAGTGTGGACGAGGG‐3′	5′‐TCAGTGTCTGAATTTACCCACC‐3′
Spata45	443 bp	5′‐GATGACTTAATATCTCACTG‐3′	5′‐GAAAGAGAGTTTATTTGTGTGTC‐3′
Gapdh	306 bp	5′‐CATGACCACAGTCCATGCCATC‐3′	5′‐TAGCCCAAGATGCCCTTCAGTG‐3′
[qRT‐PCR]			
Egr1	104 bp	5′‐GAGTCGTTTGGCTGGGATAA‐3′	5′‐GAGCGAACAACCCTATGAGC‐3′
Fos	140 bp	5′‐GGTTTCAACGCCGACTACGA‐3′	5′‐GCGCAAAAGTCCTGTGTGTT‐3′
Areg	130 bp	5′‐TGGTGAACGGTGTGGAGAAA‐3′	5′‐GATGCCGATGCCAATAGCTG‐3′
Ccdc200	151 bp	5′‐AGGCAAGGACCCCAGACTAA‐3′	5′‐TAGTTGACGTGAGTCGGGAA‐3′
Gm10337	70 bp	5′‐ACCAGATGTCAACCTTCTCAGA‐3′	5′‐CATACGACGATGTGGTGAAGA‐3′
Gm614	134 bp	5′‐TTCATGCCCAACCCCTATGC‐3′	5′‐ACTCTTGATTGTCTCCATGTTTGA‐3′
Adad2	134 bp	5′‐TGTGAAGACTGACTCCAGCG‐3′	5′‐CCTTGGCAGCCTCATAGGTC‐3′
Spata45	128 bp	5′‐AGTCCTACTGCCTGGTGGAA‐3′	5′‐GGAGGTCCTGGTACTTTCGG‐3′
Gapdh	87 bp	5′‐TGCACCACCAACTGCTTAGC‐3′	5′‐GGCATGGACTGTGGTCATGAG‐3′
U6	94 bp	5′‐CTCGCTTCGGCAGCACA‐3′	5′‐AACGCTTCACGAATTTGCGT‐3′

The completed construct, together with pPyCAG‐PBase and pPBCAG‐tTA‐IN plasmids [45, 46], was transfected into OV3121 cells using GeneJuice (Merck, Darmstadt, Germany) according to the manufacturer's protocol, and the successfully transfected cells were selected by the treatment with 0.5 mg·mL^−1^ G418 for 2 weeks. Then, the cells were spread onto 10‐cm dishes at low density and cultured for another 2 weeks. Twenty‐four colonies were picked, and two of those that were responsive to hCG were selected. As a result, OV3121‐LHR #5 and OV3121‐LHR #23 cell lines were established.

### RNA extraction and RT‐PCR analysis

Cellular and EV RNAs were extracted from cells and EVs, respectively, using ISOGEN II (Nippon Gene, Tokyo, Japan) according to the manufacturer's instructions. Total RNA was treated with TURBO DNase (Thermo Fisher Scientific), followed by DNase inactivation using DNase Inactivation Reagent (Thermo Fisher Scientific). RNA integrity was verified by agarose gel electrophoresis, and RNA concentration was measured using a NanoDrop spectrophotometer (Thermo Fisher Scientific). EV RNA samples with high integrity were used for EV RNA‐sequencing (RNA‐seq). For cellular RNA samples, 500 ng of DNase‐treated RNA was used for reverse transcription with ReverTra Ace (TOYOBO, Osaka, Japan) and oligo(dT)_20_ primers. For EV RNA samples, 100 ng of DNase‐treated RNA was reverse‐transcribed using ReverTra Ace with random primers.

PCR was performed using ExTaq polymerase (Takara Bio, Shiga, Japan) with the following cycling conditions: initial denaturation at 94 °C for 2 min, followed by 25 cycles of 94 °C for 30 s, 60 °C for 30 s, and 72 °C for 1 min. PCR products of *Lhcgr* and *Gapdh* were analyzed by agarose gel electrophoresis and visualized by ethidium bromide staining. Quantitative RT‐PCR (qRT‐PCR) was performed using an ABI 7300 Real‐Time PCR System (Thermo Fisher Scientific) with Power SYBR Green Master Mix (Thermo Fisher Scientific). The amplification conditions were 95 °C for 10 min, followed by 40 cycles of 95 °C for 15 s and 60 °C for 1 min, followed by a dissociation stage. Relative expression levels were calculated using the $2^{-\Delta \Delta C_{t}}$ method with *Gapdh* as an internal control for cellular RNAs and *U6* for EV RNAs. Primer sequences used for RT‐PCR and qRT‐PCR are listed in Table 1.

### Western blot analysis

For LHCGR detection, OV3121 cells, OV3121‐LHR #5 cells, and OV3121‐LHR #23 cells were collected and sonicated on ice in 50 mm Tris–HCl (pH 8.0) containing 1× protease inhibitor cocktail (Fujifilm Wako) and 1 mm EDTA (pH 8.0). The lysates were centrifuged at 13 000 ***g*** for 10 min at 4 °C. The resulting precipitated fractions were boiled in 50 mm Tris–HCl (pH 8.0) containing 1% sodium dodecyl sulfate (SDS) for 10 min and subsequently centrifuged at 13 000 ***g*** for 10 min. The supernatants obtained from the precipitated fractions were collected for LHCGR detection. Protein concentrations were determined using a Pierce BCA Protein Assay Kit (Thermo Fisher Scientific). Western blotting was performed as previously described [47] with minor modifications. Briefly, protein extracts were separated by 7% SDS/polyacrylamide gel electrophoresis (PAGE) and transferred onto polyvinylidene difluoride (PVDF) membranes (Fujifilm Wako). Membranes were blocked with 5% skim milk (Fujifilm Wako) in PBS for 1 h and incubated with an anti‐LHR antibody (cat no.: bs‐0984R; Bioss, Woburn, MA, USA) at a dilution of 1: 100 in PBS containing 0.1% Tween 20. Anti‐β‐Actin antibody (ACTB; Proteintech, Rosemont, IL, USA) was used as a loading control. Normal mouse IgG (Merck) was used as a negative antibody control. The signal was detected using Light Capture II (Atto, Tokyo, Japan).

For EV markers, an equal volume of the EV sample, which was prepared as in the next section, was loaded onto each well of an SDS/PAGE gel (Bio‐Rad Laboratories, Hercules, CA, USA). Ovarian extracts were prepared by homogenization in lysis buffer with BioMasher II (Nippi, Tokyo, Japan), sonication (VC‐50; Sonics & Materials, Newtown, CT, USA), and centrifugation at 12 000 ***g*** for 15 min at 4 °C. After electrophoresis, the proteins were transferred onto a PVDF membrane (Bio‐Rad Laboratories), blocked for 1 h at room temperature with Blocking Reagent for WB (Cosmobio, Tokyo, Japan), and then incubated overnight with a primary polyclonal antibody against CD63 (cat no.: 32151‐1‐AP; Proteintech Group, Rosemont, IL, USA) at a dilution of 1 : 1500, TSG101 (cat no.: 28283‐1‐AP; Proteintech) at a dilution of 1 : 1500, or Calnexin (cat no.: 10427‐2‐AP; Proteintech) at a dilution of 1 : 1500 in IMMUNO SHOT Platinum (Cosmobio). The membrane was washed with Tris buffered saline with Tween 20 (TBS‐T) and then reacted with a horseradish peroxidase‐conjugated secondary polyclonal antibody against rabbit IgG (cat no.: 7074; Cell Signaling Technology, Danvers, MA, USA) at a dilution of 1 : 3000 for 1 h. Finally, the membrane was washed again with TBS‐T. Immune complexes were detected using SuperSignal West Pico PLUS (Thermo Fisher Scientific) according to the manufacturer's protocols. The bands were visualized using a C‐DiGit Blot Scanner (LICORbio, Lincoln, NE, USA) and the Image Studio Digit software (version 5.2.5).

### Extracellular vesicle isolation

EVs were isolated from conditioned media and mouse ovaries by ultracentrifugation, modified from Doyle *et al*. and Phoonsawat *et al*. [48, 49]. Ovaries were collected 2 h after hCG or PBS injection and immediately washed with ice‐cold filtered PBS. Samples were obtained from two independent experimental batches (three and six mice per group), yielding a total of 6 and 12 ovaries per group, respectively. Ovarian tissues were homogenized on ice in 600 μL PBS. The homogenates were centrifuged at 21 600 ***g*** for 10 min to remove cells, tissue debris, and large vesicles. The supernatants were collected and diluted to a final volume of approximately 36 mL with cold PBS, followed by filtration through a 0.22‐μm membrane (Sartorius, Göttingen, Germany). Conditioned media from cultured cells were centrifuged at 900 rpm for 5 min to remove cells, followed by centrifugation at 10 000 ***g*** for 30 min to remove cell debris. The supernatant was filtered through a 0.22‐μm filter (Sartorius).

The filtrate was ultracentrifuged at 100 000 ***g*** for 70 min at 4 °C. EV pellets were washed with filtered PBS and centrifuged again at 100 000 ***g*** for 70 min. The ultracentrifugation was performed using a CP80WX ultracentrifuge equipped with a P28S swinging‐bucket rotor (Himac, Hitachinaka, Japan). EV pellets were resuspended in 200–300 μL filtered PBS.

### Transmission electron microscopy (TEM)

EV morphology was examined by transmission electron microscopy (TEM) using a modified protocol based on standard negative staining procedures. Briefly, EV suspensions (10 μL) were applied as droplets onto parafilm. Collodion‐coated TEM grids (Nissin EM, Tokyo, Japan) were pretreated to increase hydrophilicity and floated coated‐side‐down on the droplets for 1 min. Grids were washed with Milli‐Q water and negatively stained with EM stainer (Nissin EM) according to the manufacturer's instructions. TEM imaging was performed by using TEM Hitachi H‐7650 (Hitachi High‐Tech Corporation, Tokyo, Japan) at 80 kV to evaluate EV morphology and size.

For particle‐size analysis, TEM images were obtained from each experimental sample, and more than 150 individual particles were measured per sample. The numbers of particles analyzed were 167 and 178 for mouse ovarian EVs treated with hCG and PBS, respectively; 251 and 303 for OV3121‐LHR #5 EVs at 2 h; 231 and 231 for OV3121‐LHR #5 EVs at 24 h; 234 and 307 for OV3121‐LHR #23 EVs at 2 h; and 208 and 246 for OV3121‐LHR #23 EVs at 24 h, for hCG‐ and PBS‐treated samples, respectively. Particle size was measured from TEM images using the ImageJ software (National Institutes of Health, Bethesda, MD, USA). Because the EVs observed by TEM were not uniformly spherical, the maximum Feret diameter was used as the particle‐size parameter. The maximum Feret diameter was defined as the longest distance between two points along the particle boundary. Size‐distribution histograms were generated by grouping the measured particles into predefined size intervals and calculating the percentage of particles in each interval relative to the total number of particles analyzed for each sample. The proportion of particles smaller than 200 nm was also calculated for each experimental group.

### EV RNA electrophoresis and densitometric analysis

EV RNA samples were analyzed by electrophoresis to visualize changes in EV RNA profiles following hCG treatment. EV preparations were obtained from five experimental groups: OV3121‐LHR #5 cells treated with hCG or PBS for 2 and 24 h, OV3121‐LHR #23 cells treated with hCG or PBS for 2 and 24 h, and mouse ovaries treated with hCG or PBS for 2 h. Two hundred nanograms of each EV RNA were fractionated on 0.7% agarose gels and visualized by ethidium bromide staining. The gel images were analyzed using ImageJ (National Institutes of Health). Regions of interest (ROIs) corresponding to the RNA‐containing regions were defined consistently across samples. The integrated density of each ROI was measured, and background intensity was determined from an adjacent region without an RNA signal. Background‐subtracted integrated‐density values were calculated and used to compare RNA signal intensity among the experimental groups. The same image acquisition and analysis settings were applied to all samples within each experiment.

### Extracellular vesicle RNA‐sequencing (EV RNA‐seq)

EV RNA‐seq was performed using RNAs isolated from EVs derived from hCG‐ or PBS‐treated samples. For cell culture models, conditioned media of OV3121‐LHR #5 and OV3121‐LHR #23 cells were collected 24 h after treatment with hCG or PBS. A total volume of 210 mL of conditioned media per group was used for OV3121‐LHR #5 cells, while 140 mL per group was collected for OV3121‐LHR #23 cells. Conditioned media were harvested in multiple preparations of 70 mL per collection for EV isolation. EV RNA extracted from each preparation was treated with DNase and subsequently pooled within each treatment group. For ovarian samples, ovaries were collected from 18 female mice 2 h after hormone administration, including hCG‐treated (*n* = 9) and PBS‐treated groups (*n* = 9). Ovarian samples were obtained in two independent experimental batches (three and six mice per group), and EV RNAs isolated from each batch were pooled within the respective treatment group. All EV RNAs were dissolved in RNA‐free water prior to sequencing.

EV RNA libraries were prepared and sequenced on a next‐generation sequencing platform by Rhelixa, Inc (Tokyo, Japan). After ribosomal RNAs were removed, double‐strand cDNAs were synthesized, and strand‐specific libraries were prepared. The sequencing was done using the Illumina NovaSeq X Plus sequencer. Raw FASTQ files were trimmed using fastp and aligned to the reference genome using HISAT2. Read counts were generated using featureCounts in the Subread package, and gene expression levels were quantified as transcripts per million (TPM). Potential differentially expressed genes (DEGs) were selected based on expression ratios between hCG‐ and PBS‐treated groups. Genes with an upregulation ratio (hCG/PBS) ≥ 2 or a downregulation ratio (PBS/hCG) ≥ 2 were considered to be potential DEGs.

### Overexpression of DEG candidates in OV3121 cells

Coding sequences of DEG candidates (*Ccdc200*, *Gm10337*, *Gm614*, *Adad2*, and *Spata45*) were amplified by PCR using cDNA derived from a 2‐month‐old mouse testis as a template. Primer sequences are listed in Table 1. *Ccdc200*, *Gm10337*, and *Gm614* were amplified using KOD FX Neo (Toyobo), whereas *Spata45* and *Adad2* were amplified using the KAPA Polymerase Kit (KAPA Biosystems, Wilmington, MA, USA) according to the manufacturers' instructions. The pcDNA3.1(+) expression vector was linearized with EcoRV, and the PCR products were ligated into the vector. All constructs were verified by DNA sequencing.

OV3121 cells were seeded at 5.0 × 10^5^ cells in a 6‐cm dish 24 h prior to transfection. Cells were transfected with individual overexpression plasmids, empty‐vector control, or CMV‐EGFP using PEI MAX (Polysciences, Warrington, PA, USA) according to the manufacturer's instructions. Briefly, 5 μg of plasmid DNA was mixed with 5 μL of 1 mg·mL^−1^ PEI MAX in 500 μL Opti‐MEM (Thermo Fisher Scientific) and incubated for 20 min at room temperature before addition to cells. Culture medium was replaced with fresh medium prior to transfection. Cells and conditioned media were harvested 48 h post‐transfection for further analyses.

### Preparation of each DEG candidate‐enriched EVs and EV‐mediated cell number assay

EVs were isolated from conditioned media of OV3121 cells that overexpressed each gene cultured in 6‐cm dishes by differential ultracentrifugation as described above. Overexpression efficiency of each gene was validated by qRT‐PCR using gene‐specific primers in both cellular RNA and EV‐derived RNA. *Gapdh* and *U6* were used as internal controls for normalization of cellular and EV RNA levels, respectively. EV preparations derived from each candidate gene‐overexpressing group were collected separately for subsequent functional assays.

For EV‐mediated cell number analysis, OV3121 cells were seeded at 2.0 × 10^4^ cells per well in 24‐well plates 24 h prior to treatment. Cells were treated with EVs isolated from cells that were transfected with DEG‐overexpressing vectors or control vectors. The amount of EVs applied was normalized according to the culture surface area of the original 6‐cm dish to ensure equivalent EV input per well. Cell number was assessed by hemocytometer‐based cell counting for three consecutive days (D1–D3) after EV treatment. Each experiment was performed with at least three independent replicates.

## Statistical analysis

Data are presented as mean ± standard error of the mean (SEM) from at least three independent experiments. Statistical significance was evaluated using a Student's *t*‐test for comparisons between two independent groups. For comparisons among multiple groups, Tukey's multiple‐comparison test was performed. Time‐course data were analyzed by one‐way or two‐way ANOVA followed by Tukey's multiple‐comparison tests. The number of independent experiments and biological samples analyzed for each experiment is indicated in the corresponding figure legends. Differences were considered significant at *P* < 0.05. Statistical significance is denoted as follows: *P < 0.05, **P < 0.01, ***P < 0.001, and ****P < 0.0001. All statistical analyses were performed using the R software (version 4.4.2; R Foundation for Statistical Computing, Vienna, Austria).

## Results

### Establishment and validation of *Lhcgr*‐expressing OV3121 cell clones

OV3121 cells are tumor‐derived granulosa cells established from mouse ovary and are widely used as a granulosa‐cell model [44]. However, endogenous *Lhcgr* expression in OV3121 cells was low (Fig. S1), which limited their responsiveness to LH or hCG. To overcome this limitation, the ORF of mouse *Lhcgr* was introduced into OV3121 cells to establish stable granulosa‐cell models responsive to hCG stimulation. Following transfection and selection, two independent stable clones, OV3121‐LHR #5 and OV3121‐LHR #23, were established based on stable proliferation and responsiveness to hCG. RT‐PCR confirmed *Lhcgr* mRNA expression in both OV3121‐LHR #5 cells and OV3121‐LHR #23 cells (Fig. 1A). The western blot analysis further confirmed detectable LHCGR protein signals in both clones, while no signal was observed in parental OV3121 cells (Fig. 1B). These results confirmed LHCGR production in the engineered OV3121‐LHR cell models.

**Fig. 1:**
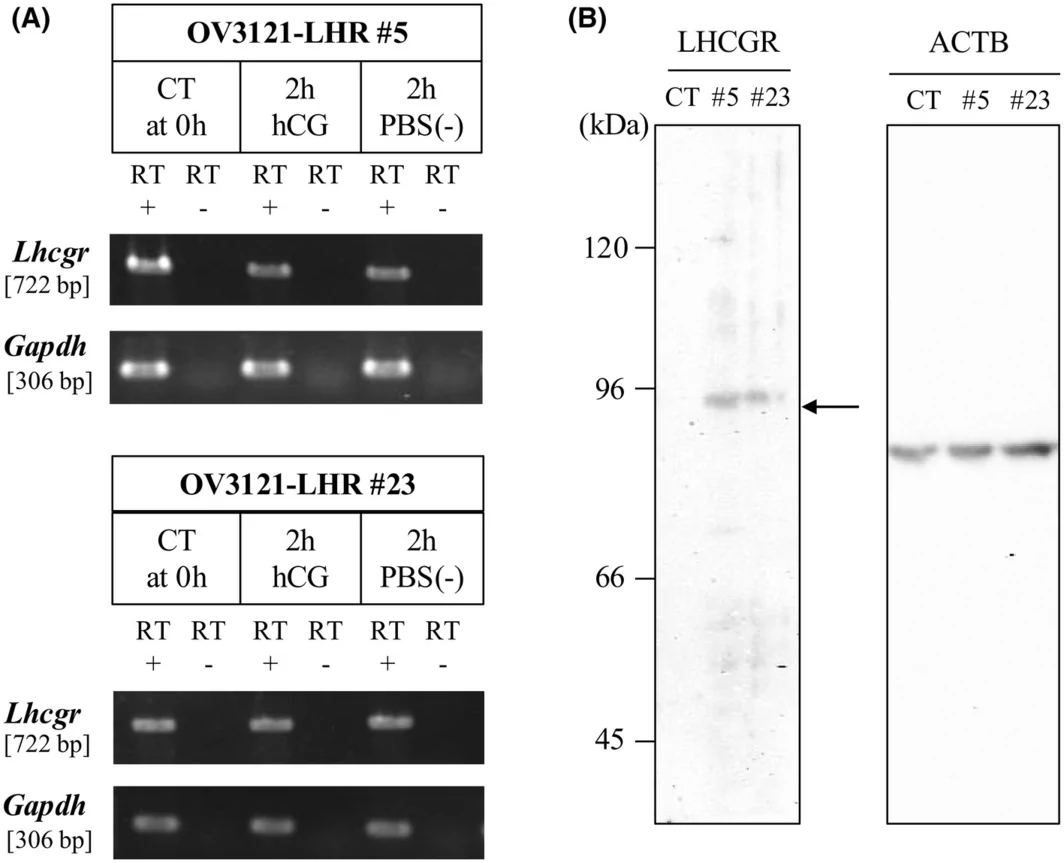
Validation of *Lhcgr* expression in OV3121‐LHR cells. OV3121‐LHR #5 cells and OV3121‐LHR #23 cells were established from mouse granulosa tumor‐derived OV3121 cells. (A) Stable expression of *Lhcgr* mRNA in OV3121‐LHR #5 cells and OV3121‐LHR #23 cells by reverse transcription polymerase chain reaction (RT‐PCR). The cells were treated with human chorionic gonadotropin (hCG) (1 IU·mL^−1^) for 2 h. Phosphate‐buffered saline (PBS)‐treated cells and 0 h samples (CT) were used as controls. Total RNAs were collected from the cells at each time point, and cDNAs were synthesized by reverse transcription with oligo(dT) primers. The PCR reaction was done for 25 cycles, and the products were visualized by agarose gel electrophoresis and ethidium bromide staining. *Gapdh* was detected as a positive control. A representative data of two experiments are shown. (B) Stable production of luteinizing hormone/choriogonadotropin receptor (LHCGR) protein in OV3121‐LHR cells by western blot analysis. Insoluble fraction was prepared from OV3121‐LHR #5 cells (#5), OV3121‐LHR #23 cells (#23), and their parental OV3121 cells (CT) by sonication and lysis with detergent. The proteins were separated by sodium dodecyl sulfate/polyacrylamide gel electrophoresis (SDS/PAGE), transferred to a membrane, and used for detection of LHCGR protein with its specific antibody. The LHCGR signal was detected at approximately 90 kDa in both OV3121‐LHR clones (#5 and #23), whereas no corresponding band was detected in parental OV3121 cells (CT). β‐Actin (ACTB) was detected as an internal control. The positions of molecular mass markers are indicated on the left. A representative data of two experiments are shown.

OV3121‐LHR cells were treated with 1 IU·mL^−1^ hCG or PBS for 0–2 h, and cells were collected for RT‐PCR and qRT‐PCR analyses. Expression of *Lhcgr* was clearly detected in both OV3121‐LHR clones by RT‐PCR after the treatment with hCG and PBS (Fig. 1A). To evaluate the responsiveness to hCG stimulation, the expression of the immediate‐early genes, *Egr1* and *Fos*, known downstream targets of LHCGR signaling, was examined by qRT‐PCR. Both OV3121‐LHR #5 cells and OV3121‐LHR #23 cells showed significant induction of *Egr1* and *Fos* following hCG treatment at 2 h compared with PBS‐treated cells and 0 h controls (Fig. 2A,B), indicating that the introduced receptor was functionally responsive to hCG. We further examined the expression of the ovulatory‐associated genes *Areg*, *Ptgs2*, and *Ereg* [18, 19], and *Areg* expression was significantly higher at 2 h after hCG treatment than at 0 h and in the corresponding PBS‐treated controls (Fig. 2C). In contrast, *Ptgs2* and *Ereg* were not detectable in either OV3121‐LHR clone (Data not shown). These results confirmed the successful establishment of *Lhcgr*‐expressing OV3121 cell models that showed transcriptional responses to hCG stimulation, although properties of these clones might not be the same as granulosa cells.

**Fig. 2:**
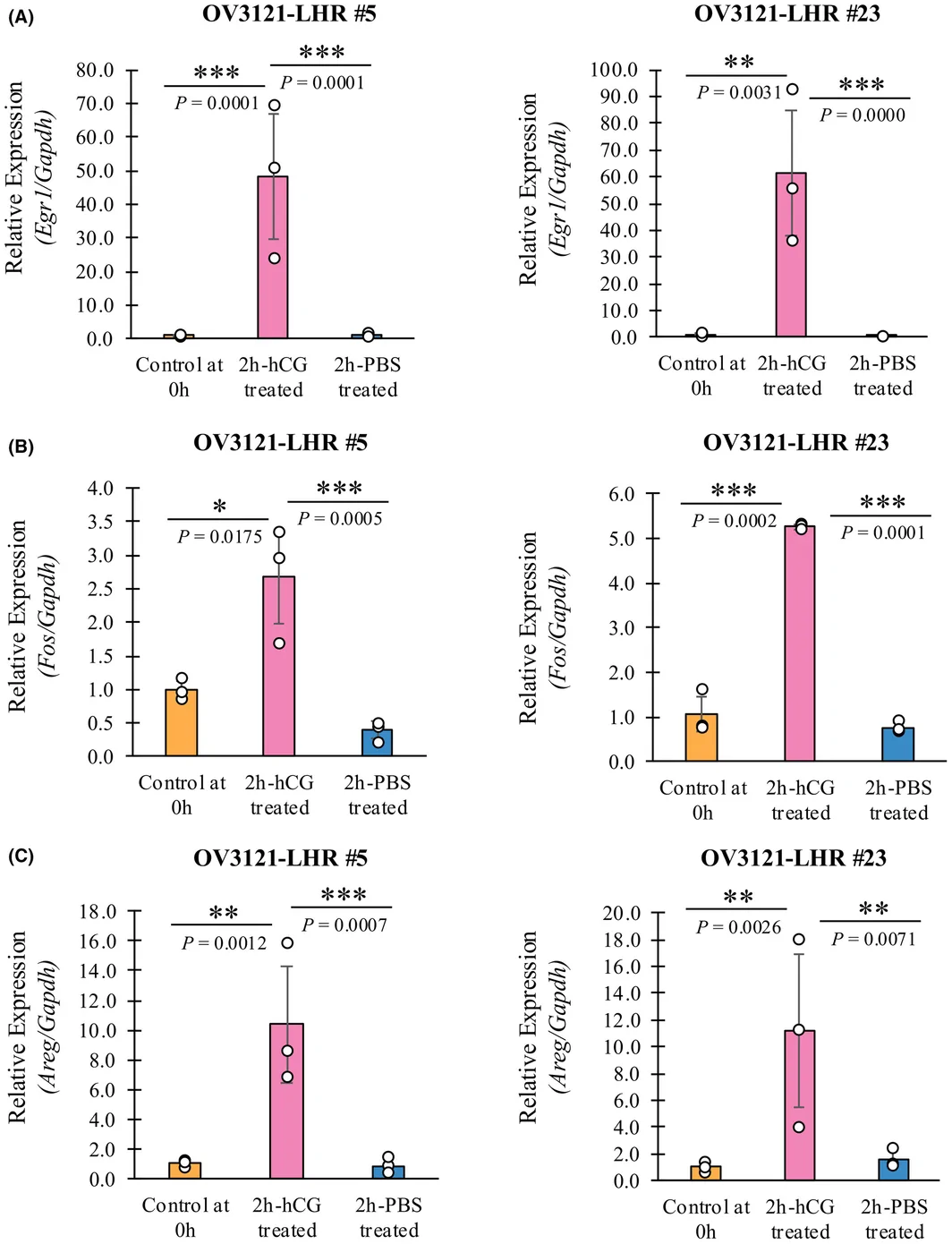
Responsiveness of OV3121‐LHR cells to human chorionic gonadotropin (hCG) stimulation by quantitative reverse transcription polymerase chain reaction (qRT‐PCR). OV3121‐LHR #5 cells and OV3121‐LHR #23 cells were treated with hCG (1 IU·mL^−1^) or phosphate‐buffered saline (PBS) for 2 h. The cells before the hormone treatment were also collected as a 0‐h control. Total RNAs were purified from each sample, and cDNAs were synthesized with oligo(dT) primers. Relative mRNA levels of *Egr1* (A), *Fos* (B), and *Areg* (C) were determined by quantitative PCR, normalizing to *Gapdh*, an internal control, using the $2^{-\Delta \Delta C_{t}}$ method. Data are presented as means ± standard error of the mean (SEM) from three independent experiments (*n* = 3). Statistical analysis was performed using Tukey's test, and *P* values are indicated within the graph. *P* < 0.05 was considered statistically significant (*P < 0.05, **P < 0.01, ***P < 0.001).

### Characterization of EV preparations from OV3121‐LHR cells and mouse ovaries

To determine whether hCG stimulation affects EV production and cargo composition, EVs were isolated from conditioned media of OV3121‐LHR #5 and OV3121‐LHR #23 cells following hCG or PBS treatment for 2 and 24 h and from mouse ovaries at 2 h after hCG administration. In total, five experimental groups were collected, and the characteristics of these EV preparations were validated.

TEM revealed vesicles displaying characteristics of EV morphology across all experimental groups, including a lipid bilayer membrane, spherical shape with smooth contours (Fig. 3A). Quantitative particle measurements were performed from TEM images using the maximum Feret diameter in ImageJ. More than 150 particles were analyzed for each experimental sample. Particles measuring 50–75 nm accounted for the highest proportion in every sample (Fig. 3B). The proportions of particles < 200 nm were 96.41% and 98.35% for hCG‐ and PBS‐treated OV3121‐LHR #5 samples at 2 h, respectively; 97.84% and 94.81% for OV3121‐LHR #5 at 24 h; 95.73% and 99.02% for OV3121‐LHR #23 at 2 h; 98.08% and 99.59% for OV3121‐LHR #23 at 24 h; and 91.02% and 96.63% for mouse ovaries at 2 h (Fig. 3B). As a result, more than 90% of measured particles were smaller than 200 nm across all samples (Fig. 3C). These measurements provide quantitative information on the size distribution of the vesicular structures recovered from the different experimental preparations.

**Fig. 3:**
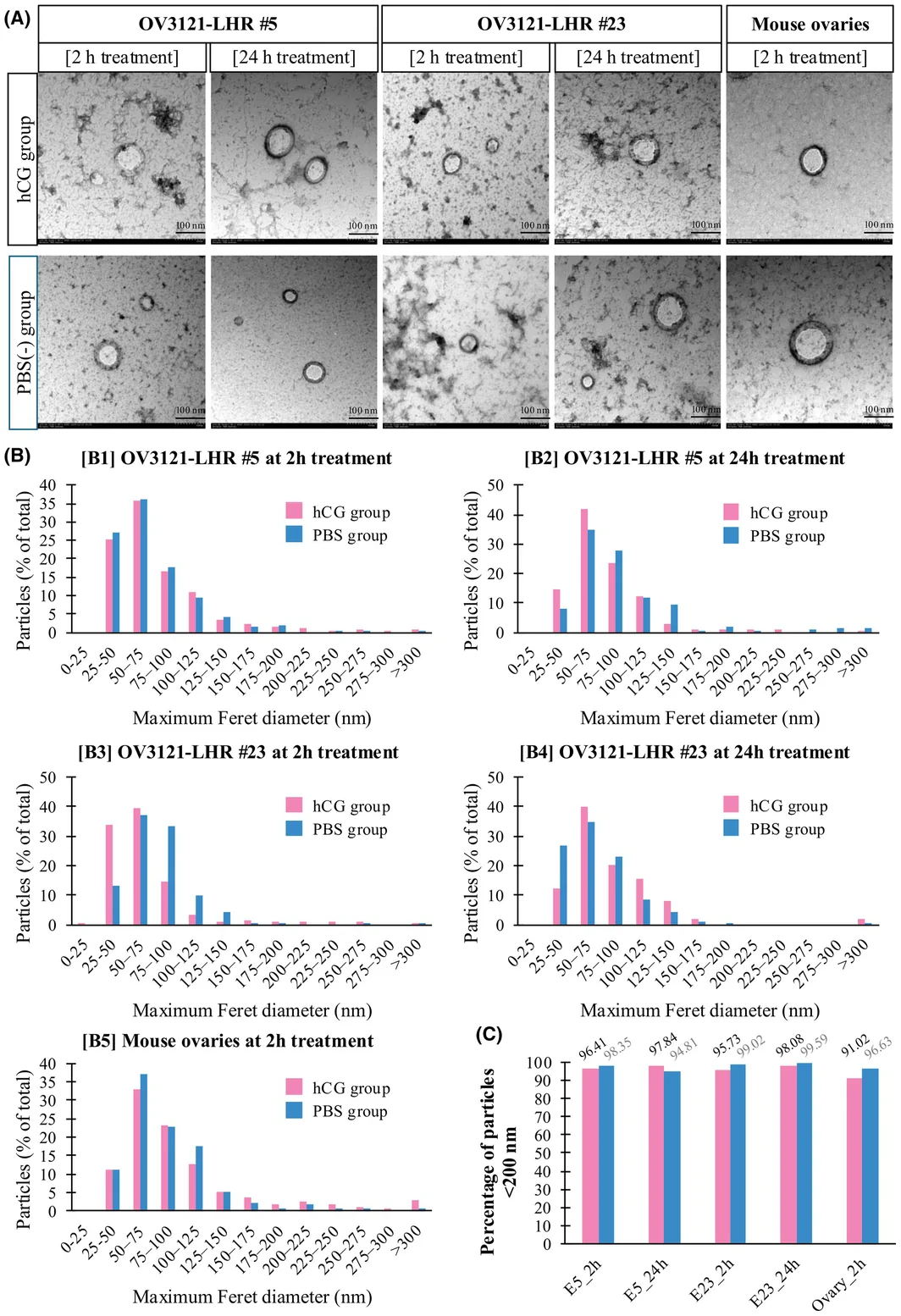
Morphological characterization of human chorionic gonadotropin (hCG)‐induced extracellular vesicles (EVs) by transmission electron microscope (TEM). OV3121‐LHR #5 cells and OV3121‐LHR #23 cells were treated with human chorionic gonadotropin (hCG) (1 IU·mL^−1^) for 2 and 24 h, with phosphate‐buffered saline (PBS)‐treated cells serving as controls. On the other hand, immature mice were primed with serotropin for 48 h followed by hCG or PBS injection, and ovaries were collected 2 h later. EVs were isolated from conditioned media for OV3121‐LHR cells and from homogenates for ovaries at the indicated time points by the method using ultracentrifugation. The isolated EVs were analyzed by TEM. (A) TEM revealed the presence of vesicles with typical EV morphology, including a spherical shape, lipid bilayer structure, and a diameter of 25–150 nm in all experimental groups. Scale bar, 100 nm. EVs were collected from 60‐mL conditioned medium of OV3121‐LHR #5 cells at 2 and 24 h after hCG/PBS treatment, 60‐mL conditioned medium of OV3121‐LHR #23 cells at 2 and 24 h after hCG/PBS treatment, and 14 ovaries at 2 h after hCG/PBS injection. A representative photograph in each experimental sample is shown here. (B) Particle‐size‐distribution histograms generated from TEM images. Particle size was determined as the maximum Feret diameter using ImageJ. The *x*‐axis represents particle size (0–300 nm), and the *y*‐axis represents the percentage of measured particles within each size interval (% of total measured particles). More than 150 particles were measured for each experimental sample. The majority of measured particles were distributed within the 25–150 nm range. The pink bar represents the hCG‐treated samples, whereas the blue bar represents the PBS‐treated samples. (C) Percentage of measured particles smaller than 200 nm in each experimental group. Particles smaller than 200 nm accounted for 91.02–99.59% in the indicated samples. The pink bar represents the hCG‐treated samples, whereas the blue bar represents the PBS‐treated samples.

The western blot analysis was performed to further characterize EV preparations. Here, we examined three experimental groups: OV3121‐LHR #5 at 24 h, OV3121‐LHR #23 at 24 h, and mouse ovaries at 2 h. CD63 and TSG101 are EV‐associated proteins, and our Western results confirmed the presence of both proteins in all EV samples (Fig. 4A,B). Calnexin, an intracellular endoplasmic reticulum protein, was also investigated, and the signal was observed in EVs from ovaries but not from OV3121‐LHR cells (Fig. 4C). Together, the TEM morphology, particle‐size distribution, and EV‐associated protein profiles support the characterization of the isolated preparations as EV‐containing fractions although there was some contamination from the intracellular membrane in the ovarian sample.

**Fig. 4:**
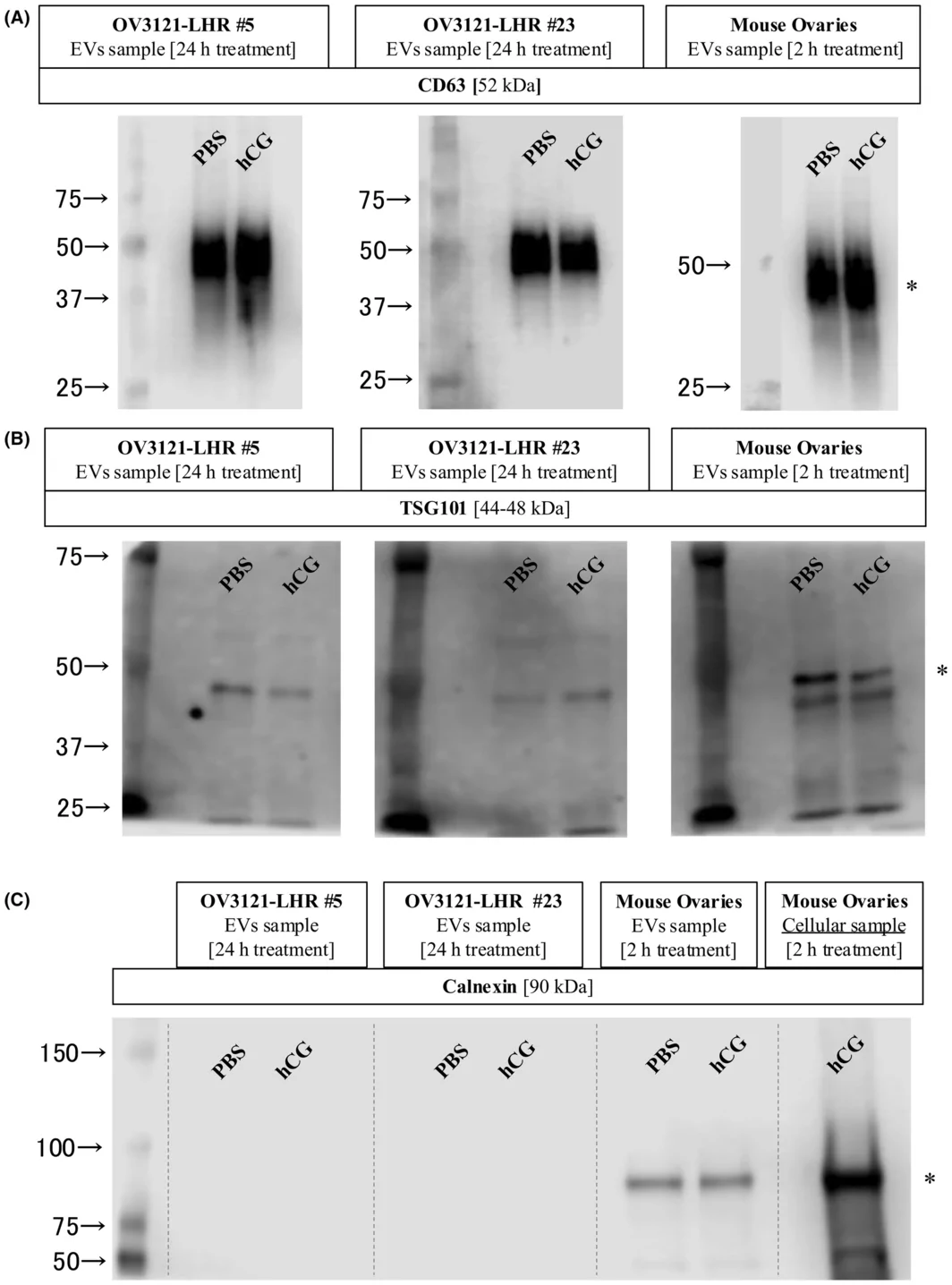
Detection of extracellular vesicle (EV)‐related protein markers by western blot analysis. EV‐enriched preparations were isolated from 70 mL of conditioned medium from OV3121‐LHR #5 cells and OV3121‐LHR #23 cells treated with human chorionic gonadotropin (hCG) (1 IU·mL^−1^) or phosphate‐buffered saline (PBS) for 24 h. For the mouse ovarian model, immature female mice were primed with serotropin (5 IU) for 48 h and subsequently with hCG (5 IU) or PBS. Whole ovaries were collected 2 h after treatment, and EV‐enriched preparations were obtained from pooled ovarian samples (five mice per group). Protein extracts from the whole ovary were prepared from one adult mouse per treatment group. These EV‐enriched and tissue protein preparations were separated by sodium dodecyl sulfate/polyacrylamide gel electrophoresis (SDS/PAGE), transferred to a membrane, and used for detection with each antibody. Representative data from one (A), three (B), and two experiments (C) are shown for each experimental model. (A) Detection of CD63, an EV‐associated marker, in EV‐enriched preparations from the indicated experimental groups. CD63 immunoreactive bands were detected at approximately 52 kDa. (B) Detection of TSG101, another EV‐associated marker, in EV‐enriched preparations from the indicated experimental groups. TSG101 immunoreactive bands were detected at approximately 44–48 kDa. (C) Detection of calnexin, an endoplasmic reticulum‐associated intracellular protein, in the indicated samples. Calnexin was not detected in EV‐enriched preparations from OV3121‐LHR #5 cells or OV3121‐LHR #23 cells under either hCG‐ or PBS‐treated conditions but was detected at approximately 90 kDa both in EV‐enriched preparations from ovaries and in the ovary extract. These results support the successful isolation of EV samples though intracellular membranes were contaminated in EVs from ovaries.

### hCG stimulation is associated with changes in EV RNA profiles in OV3121‐LHR cells and mouse ovaries

To assess whether hCG modulates EV RNA cargo, EV RNA composition was compared between hCG‐ and PBS‐treated groups by agarose gel electrophoresis (Fig. 5A). EV RNA profiles seemed to differ between hCG‐ and PBS‐treated preparations across the experimental models. To provide quantitative support for the observed differences, the RNA electrophoresis images were analyzed by densitometry using ImageJ (Fig. 5B). Integrated‐density measurements were obtained from defined RNA‐containing regions after background subtraction. For EV RNAs from OV3121‐LHR cells, the highest peak shifted from high‐molecular weight to low‐molecular weight by the hCG treatment (Fig. 5B1–B4). In contrast, EV RNAs from ovaries showed a shift of the highest peak from low‐molecular weight to high‐molecular weight by the hCG treatment (Fig. 5B5). The densitometric profiles demonstrated differences in RNA signal patterns between hCG‐ and PBS‐treated preparations. These results suggest that hCG stimulation alters the RNA composition of EVs in both OV3121‐LHR cells and mouse ovaries.

**Fig. 5:**
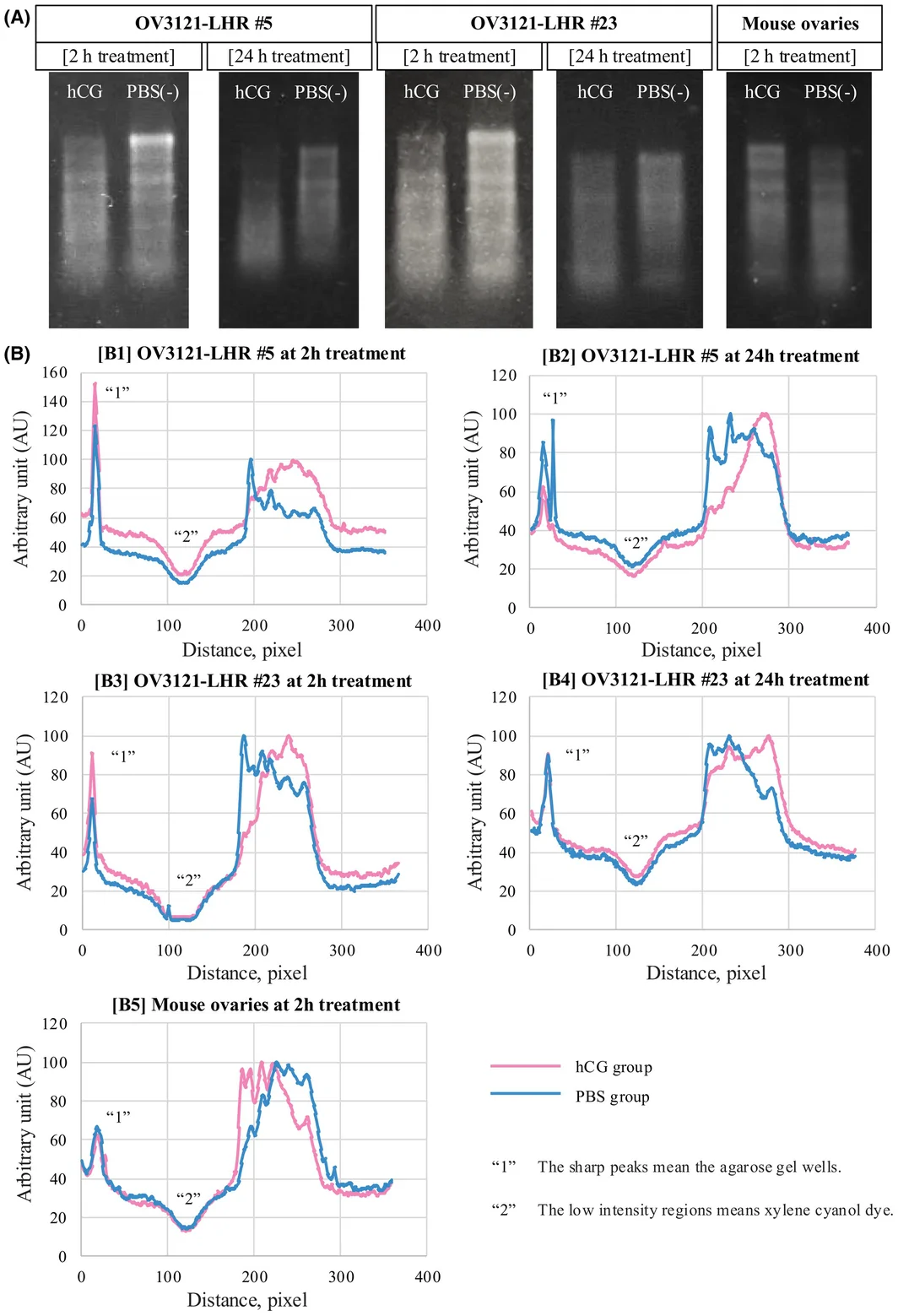
Changes in extracellular vesicle (EV)‐associated RNA profiles following human chorionic gonadotropin (hCG) treatment. EVs were isolated from conditioned media of OV3121‐LHR #5 cells and OV3121‐LHR #23 cells treated with hCG (1 IU·mL^−1^) or phosphate‐buffered saline (PBS) for 2 or 24 h. For the mouse ovarian model, immature female mice were primed with serotropin (5 IU), followed 48 h later by administration of hCG (5 IU) or PBS. Whole ovaries were collected 2 h after hCG or PBS treatment, and EVs were isolated. Total RNAs were extracted from these samples and analyzed by electrophoresis on 0.7% agarose gels. Representative images of at least three experiments for each experimental model are shown. (A) Representative agarose gel electrophoresis images of EV‐associated RNAs from the indicated experimental groups. Approximately 200 ng of EV‐associated RNA was loaded per sample. Differences in the intensity and distribution of low‐ and high‐molecular‐weight RNA signals were observed between hCG‐ and PBS‐treated samples. (B) Densitometric profiles of EV‐associated RNA electrophoresis. Signal intensity was quantified from the agarose gel images using ImageJ. The pink line represents the hCG‐treated samples, and the blue line represents the PBS‐treated samples. Sharp peaks ‘1’ correspond to the positions of the agarose gel wells, and low‐intensity regions ‘2’ correspond to the migration of xylene cyanol dye. The densitometric profiles were used to compare the distribution and relative intensity of RNA signals between hCG‐ and PBS‐treated samples, and peak shifts were observed in all samples following the hormone treatment.

### Identification of potential hCG‐responsive EV RNAs common to OV3121‐LHR cells and mouse ovaries

EV RNA‐seq was performed using EV RNAs isolated from three experimental models: OV3121‐LHR #5 cells (24 h), OV3121‐LHR #23 cells (24 h), and mouse ovaries (2 h) following hCG or PBS treatment (Fig. S2). For OV3121‐LHR cells, EV RNA was obtained from multiple EV preparations derived from conditioned media and pooled within each treatment group, with at least two biological replicates included for sequencing analysis. For mouse ovaries, EV RNA was isolated from ovaries collected from two independent experimental batches of mice (3 and 6 mice per group), and RNA samples were pooled within each treatment group prior to sequencing. Because the amount of EV RNA available from individual preparations was limited, pooling was necessary to obtain sufficient RNA for library preparation and sequencing.

Differential expression analysis was performed based on TPM expression ratios between hCG‐ and PBS‐treated groups. Transcripts with an upregulation ratio (hCG/PBS ≥ 2) or a downregulation ratio (PBS/hCG ≥ 2) were defined as potential DEGs. As a result, thousands of RNAs were found to be potential DEGs in each experimental model, and 111 and 122 genes were identified as commonly upregulated and downregulated RNAs, respectively, shared among the three experimental models (Fig. 6A, Tables S1 and S2). To visualize the expression patterns of these common potential DEGs, their relative expression levels were displayed as a heatmap based on row‐scaled Z‐scores calculated from log_2_(TPM + 1) values (Fig. 6B). The heatmap showed clear clustering separation between hCG‐ and PBS‐treated samples across all experimental groups.

**Fig. 6:**
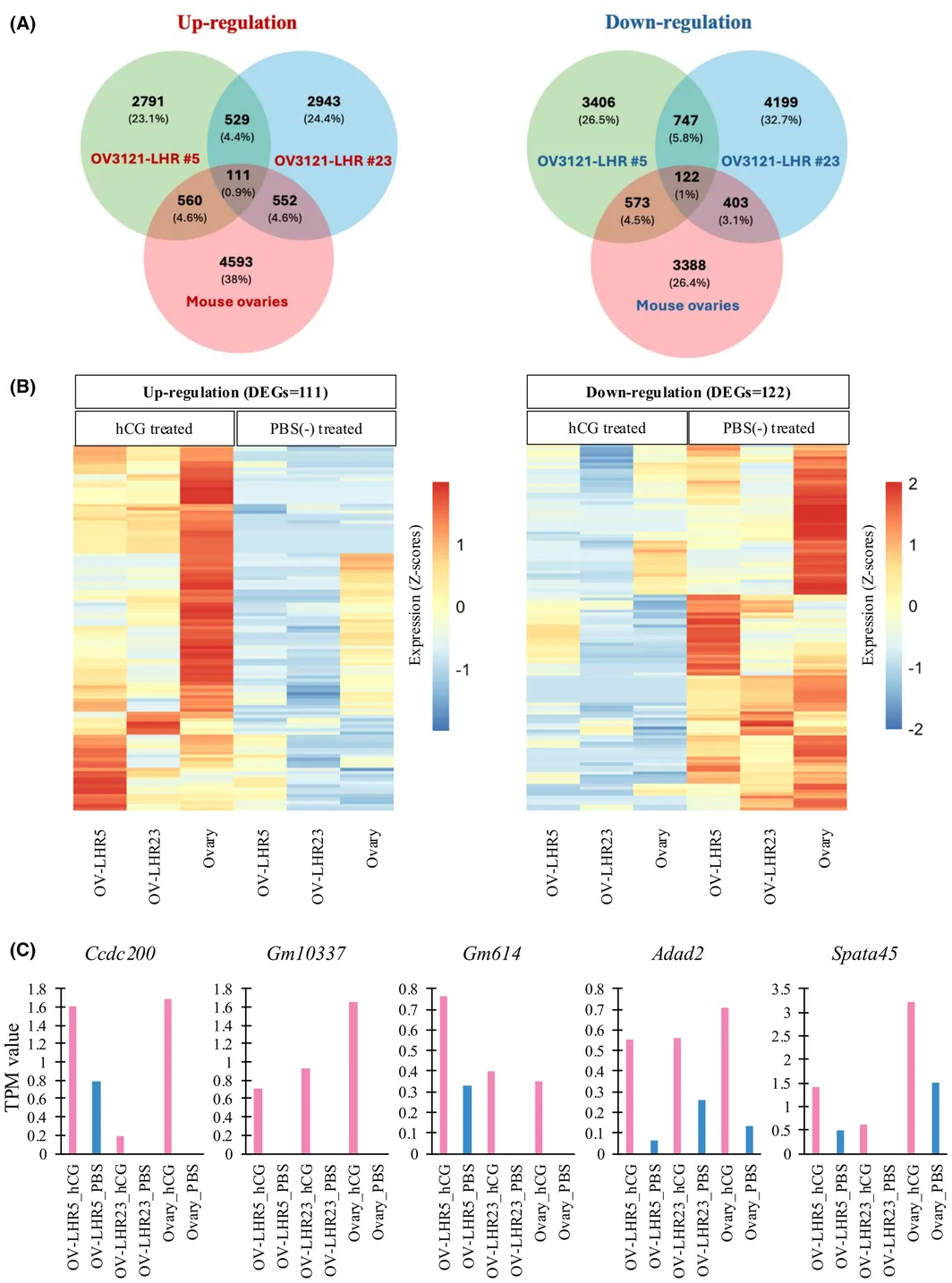
Identification of potential differentially expressed genes (DEGs) within extracellular vesicles (EVs) in response to human chorionic gonadotropin (hCG) in OV3121‐LHR cells and mouse ovaries. EV RNAs were isolated from OV3121‐LHR #5 cells, OV3121‐LHR #23 cells, and mouse ovaries that were treated with hCG or phosphate‐buffered saline (PBS). A total of 210 mL and 140 mL culture media per treatment group was collected from OV3121‐LHR #5 cells and OV3121‐LHR #23 cells, respectively, in multiple 70‐mL preparations. For the ovarian model, a total of 18 mice (nine per group) were used in two independent experimental batches (three and six mice per group). EVs were isolated from these samples, and total RNAs were purified and treated with Turbo DNase. These RNAs were pooled for each experimental sample and subject to RNA‐seq analysis. (A) Venn diagram illustrating the number of upregulated (left) and downregulated genes (right) in EVs from each sample. Thousands of genes were changed in each sample, and 111 and 122 genes were found to be commonly upregulated and downregulated EV RNAs, respectively, shared among the three models. (B) Heatmap showing row‐scaled *Z*‐scores calculated from log_2_(TPM + 1) values of the 111 upregulated (left) and 122 downregulated EV RNAs (right) comparing hCG‐treated and PBS control groups across experimental conditions. (C) Bar graphs showing raw transcripts per million (TPM) values of five selected protein‐coding genes (*Ccdc200, Gm10337, Gm614, Adad2*, and *Spata45*) identified as potentially upregulated EV RNAs common to all of OV3121‐LHR #5 cells, OV3121‐LHR #23 cells (24 h after hCG treatment), and mouse ovaries (2 h after hCG treatment).

Further analysis focused on the commonly upregulated RNAs. The 111 commonly upregulated transcripts were further prioritized based on their expression abundance in hCG‐treated samples. Because the overall TPM distributions differed among the three experimental models, dataset‐specific minimum TPM thresholds were applied. A minimum TPM value of 0.35 was used for OV3121‐LHR #5 cells and mouse ovaries, whereas a threshold of 0.19 was applied to OV3121‐LHR #23 cells. Transcripts meeting the predefined TPM criteria across the respective experimental models were retained, resulting in 27 candidate upregulated EV RNA transcripts (Table S3).

The 27 candidate transcripts were then classified according to their gene biotypes, such as protein‐coding gene, lncRNA, and pseudogene (Table S3). For subsequent exploratory functional evaluation, we focused on protein‐coding transcripts for which gene annotations and experimental sequence information were available. This annotation‐based selection resulted in five genes: *Ccdc200, Gm10337, Gm614, Adad2*, and *Spata45* (Table S4). The TPM value levels of these genes are shown in Fig. 6C, where higher expression was observed in the hCG‐treated groups than in PBS‐treated controls across the three experimental models.

Collectively, these findings identified a conserved subset of protein‐coding EV RNA candidates that were consistently enriched following hCG stimulation in both OV3121‐LHR cells and mouse ovary.

### EVs enriched with hCG‐induced RNA candidates increase ovarian‐cell number

To investigate the functional roles of the five protein‐coding RNAs (*Ccdc200*, *Gm10337*, *Gm614*, *Adad2*, and *Spata45*) within hCG‐stimulated EVs, each gene was overexpressed in OV3121 cells. Successful overexpression was confirmed by qRT‐PCR using cellular RNAs for all genes (Fig. 7A). Moreover, all RNAs were significantly enriched in EVs derived from these potential DEG‐overexpressing cells, compared with empty‐vector controls (Fig. 7B), demonstrating efficient production and incorporation of these transcripts into EVs.

**Fig. 7:**
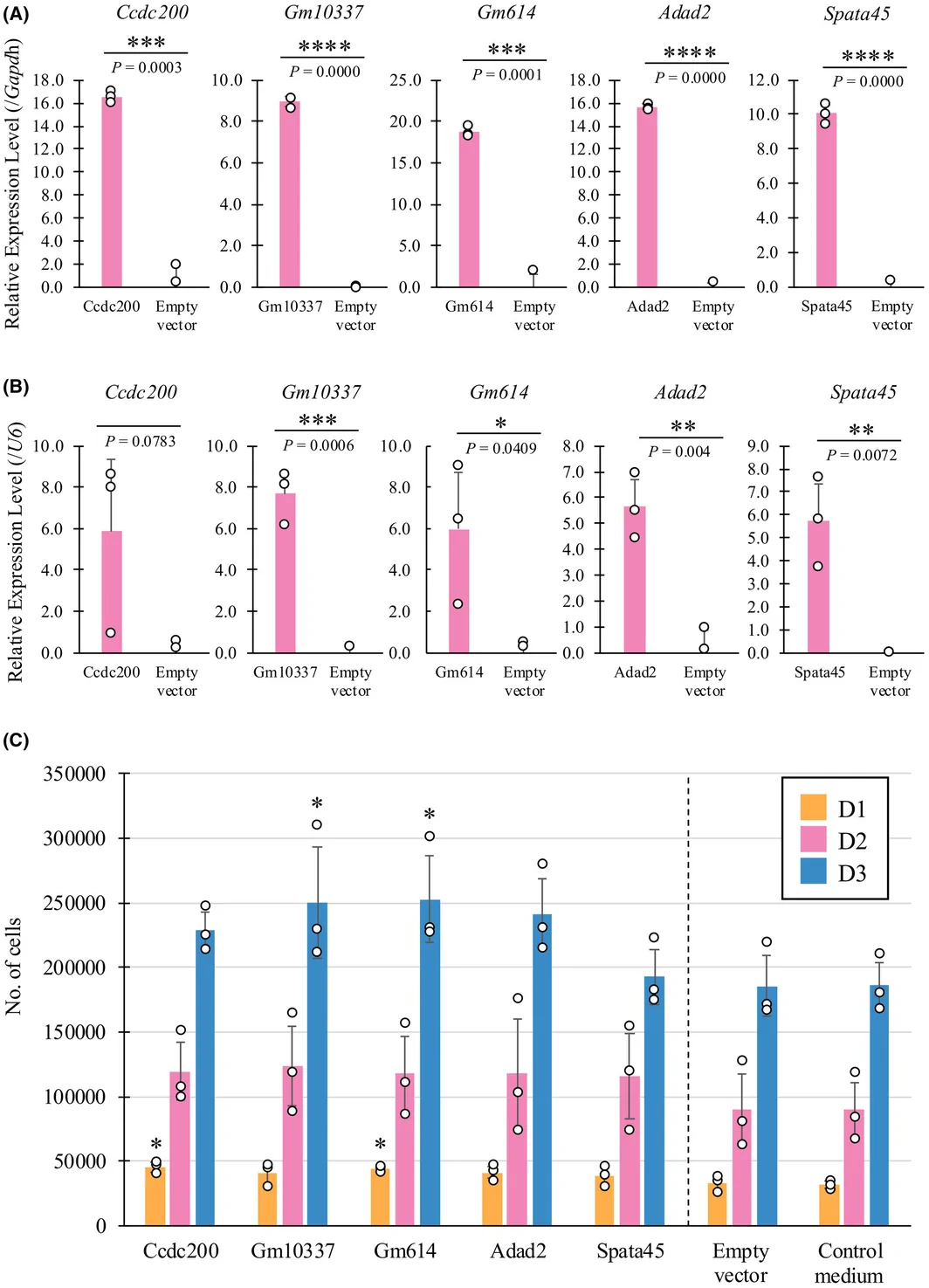
Extracellular vesicles (EVs) enriched with human chorionic gonadotropin (hCG)‐induced RNA candidates increase ovarian‐cell number. OV3121 cells were transfected with expression constructs encoding each of five EV RNA candidates to generate potential differentially expressed gene (DEG)‐overexpressing cells. Three days after transfection, cells and conditioned media were collected separately for RNA analysis and EV isolation. To assess functional effects, EVs isolated from potential DEG‐overexpressing cells were applied to culture media of OV3121 cells, and the cell number was monitored for 3 days. (A, B) Overexpression of each potential DEG in its overexpressing cells (A) and enrichment of corresponding transcripts in EVs (B) were confirmed by quantitative reverse transcription polymerase chain reaction (qRT‐PCR). Cellular mRNA levels were normalized to *Gapdh*, and EV RNA levels were normalized to *U6*, using the $2^{-\Delta \Delta C_{t}}$ method. Data are presented as mean ± standard error of the mean (SEM) from three independent biological replicates (*n* = 3). Statistical significance was evaluated using Student's *t*‐test. *P* < 0.05 was considered statistically significant (*P < 0.05, **P < 0.01, ***P < 0.001, ****P < 0.0001). (C) Time‐course effects of potential DEG‐overexpressing EVs on OV3121 cell number from Day 1 to Day 3 (D1–D3). Recipient OV3121 cells were seeded at 2.0 × 10^4^ cells per well in 24‐well plates and treated 24 h later with EV preparations from candidate RNA‐overexpressing donor cells or empty‐vector control cells. The amount of EV preparation added to each well was normalized according to the culture surface area of the donor 6‐cm dishes to maintain a comparable EV input among experimental groups. Cell numbers were determined using a hemocytometer on Days 1, 2, and 3 (D1–D3) after EV treatment. Cells treated with EVs containing overexpressed *Ccdc200*, *Gm10337*, and *Gm614* mRNAs exhibited significantly increased the cell number compared with empty vector–containing EVs. Data are presented as means ± SEM (*n* = 3) from three independent biological replicates (*n* = 3). Statistical analysis was performed using two‐way ANOVA followed by Tukey's test. *P* < 0.05 was considered statistically significant (*P < 0.05).

To determine whether EVs carrying hCG‐responsive RNA candidates could have some effects on granulosa cells, EVs from potential DEG‐overexpressing OV3121 cells were applied to OV3121 recipient cells, and the cell number was counted over 3 days (D1–D3) (Fig. 7C). On Day 1 (D1), EVs derived from *Ccdc200*‐ and *Gm614*‐overexpressing cells significantly increased cell numbers compared with empty‐vector controls. No significant differences were observed on Day 2 (D2). By Day 3 (D3), EVs enriched with *Gm10337* and *Gm614* showed significantly higher cell numbers than controls. Overall, EVs containing hCG‐induced RNA candidates, including *Adad2* and *Spata45*, exhibited an upward trend in cell number compared with the empty‐vector group, whereas control medium alone did not differ from empty vector–derived EVs. These results suggest that specific hCG‐induced EV RNA candidates, particularly *Gm614*, contribute to enhanced ovarian‐cell proliferation or cell survival.

## Discussion

EVs have emerged as important mediators of intercellular communication within the ovarian follicle [28, 50, 51, 52]. In the present study, we demonstrated that hCG stimulation altered the RNA cargo of EVs derived from OV3121‐LHR cells and mouse ovaries and identified a subset of EV‐associated transcripts that were consistently regulated across these models following the hormone stimulation. Exploratory RNA sequencing identified 111 commonly upregulated and 122 commonly downregulated transcripts, from which 27 upregulated candidates were prioritized and five transcripts (*Ccdc200*, *Gm10337*, *Gm614*, *Adad2*, and *Spata45*) were selected for further evaluation. Functional analysis suggests that EVs associated with *Ccdc200*, *Gm10337*, and *Gm614* overexpression increased the recipient OV3121 cell number at specific time points. These findings identify EV‐associated RNA candidates responsive to hCG stimulation and highlight a potential role of EV‐mediated RNA transfer in coordinating ovarian cellular responses to hCG.

It is well known that miRNAs are enriched in EVs within follicular fluid, and their involvement in some diseases, such as polycystic ovary syndrome and the possibility of their application to therapy or assisted reproductive technology, have been discussed [31, 32, 33, 34, 35, 36]. The studies reported the profiles of EV miRNAs and their dynamic changes during follicular development as well as the possible function before and after LH surge or hCG stimulation. The profile was also reported for long RNAs of ovarian EVs before LH surge, suggesting their association with metabolic pathways and epigenetic regulation [37, 38]. In the current study, we revealed that hCG could change the profile of long RNAs within EVs from ovaries and cultured granulosa cell‐derived models, which is consistent with the dynamic change in proteome by hCG in a human granulosa‐cell line [41]. Although the profiles among ovaries and two cell models were not necessarily similar, our data provide the foundation for elucidating the physiological significance of hCG‐associated EV RNAs and for future applications.

To examine hCG‐responsive EV RNA changes in granulosa cells, we engineered OV3121 cells and established two stable lines that constitutively expressed the LH receptor, LHCGR. OV3121 cells originate from mouse granulosa‐cell tumors and partially retain granulosa cell‐like characteristics [44, 53, 54]. However, their endogenous *Lhcgr* expression was low, and the introduction of this receptor gene was necessary to restore responsiveness to hCG stimulation. Rapid induction of the immediate‐early genes, *Egr1* and *Fos*, as well as the EGF‐like factor, *Areg*, following hCG stimulation confirmed that the hCG signaling pathway could function in both OV3121‐LHR clones [18, 55]. Importantly, the establishment and usage of two stable clones helped us minimize potential clonal variation. However, *Ptgs2* and *Ereg*, two genes downstream of hCG, were not expressed in our OV3121‐LHR cells, indicating that these cells do not fully reproduce the transcriptional response of normal preovulatory granulosa cells [44, 53]. The tumor‐derived origin of OV3121 cells might also affect different characteristics of our OV3121‐LHR cell models from *in vivo* granulosa cells. Despite these limitations, the OV3121‐LHR model provided a stable system for examining hCG‐induced responses in granulosa cells.

Interestingly, electrophoretic analysis of EV RNAs revealed a shift toward lower molecular weight species in hCG‐treated OV3121‐LHR cells, a pattern that was not observed in EVs derived from whole ovarian tissue. While EVs are known to contain diverse RNA populations involved in intercellular communication [56, 57, 58, 59, 60], the present data may suggest that hCG stimulation preferentially enriches smaller RNA species in a cell type–dependent manner. The discrepancy between OV3121‐LHR models and ovarian samples may reflect differences in cellular composition and physiological complexity, as the ovary comprises multiple interacting cell types and extracellular factors that could mask or dilute EV RNA composition in a single cell type. OV3121‐LHR models may exhibit a more direct or simplified response to hCG stimulation, which might result in different EV RNA size distribution. In the ovarian context, EVs within follicular fluid mediate communication among granulosa cells, theca cells, and oocytes [28, 50], suggesting that EV RNA composition is tightly regulated *in vivo*. Thus, the simplified response to hCG in EVs from OV3121‐LHR cells might be modulated in the intact ovarian environment. Alternatively, the difference might be due to the tumor cell characteristics of OV3121 cells, or the contamination of intracellular membranes in our ovarian EV preparations might have some effects on RNA composition. In any case, these findings support the notion that hCG stimulation alters the composition of EV RNAs in ovarian granulosa cells.

Consistent with differences in electrophoretic patterns, RNA‐seq analysis revealed that thousands of EV RNAs were changed in individual models. However, only a limited subset of transcripts was consistently altered across the three experimental models, and expression levels of those shared transcripts varied among OV3121‐LHR #5 cells, OV3121‐LHR #23 cells, and mouse ovary samples. Such variation may reflect differences in cellular composition, EV production, RNA loading, or the tissue microenvironment across the three experimental models. For ovary samples, the ovarian microenvironment contains multiple interacting cell populations and signaling factors that may influence EV production and RNA packaging [50, 57]. Therefore, despite variability among models, the shared EV RNAs provide a basis for prioritizing candidate hCG‐responsive transcripts that are observed across different experimental contexts.

From the commonly upregulated EV RNAs, five protein‐coding transcripts (*Ccdc200, Gm10337, Gm614, Adad2*, and *Spata45*) were selected for further examination. *Adad2* and *Spata45* are primarily characterized as testis‐enriched genes involved in germ cell development [61, 62, 63, 64], and their roles in the ovary remain largely undefined, suggesting potential context‐dependent functions. *Gm10337* remains poorly characterized, particularly in ovarian cells. In contrast, coiled‐coil domain proteins, including the *Ccdc* family, are involved in cytoskeletal organization, intracellular transport, and cell division, implicating *Ccdc200* in proliferative or structural regulation in granulosa cells [65].

Among the five genes, *Gm614* is of particular interest, as it has been shown to suppress apoptotic signaling, thereby promoting cell survival [66]. This function is consistent with the observed increase in OV3121 cell number following treatment with EVs containing *Gm614* transcript because the increase might be due to the enhanced cell survival effect by protecting cells from cell death events, such as apoptosis. This is also consistent with the fact that granulosa cells stop their proliferation after the LH surge [15, 16]. If our experimental results reflect the events after the LH surge, EVs with overexpressed *Gm614* might function in granulosa‐cell survival. Alternatively, if the results reflect the indirect action on granulosa cells before the LH surge, EVs with Gm614 might promote cell proliferation. *Gm10337* and *Ccdc200*, the other transcripts that showed significant effects, may also be involved in the regulation of cell proliferation or cell survival. However, since OV3121 cells are derived from the tumor, it is also possible that they promote tumor growth rather than regulate ovarian cells. Moreover, overexpression of a particular RNA in EVs might change the entire EV cargo, and it is not appropriate to attribute the observed effect to the candidate RNAs alone. Given the relatively low abundance of individual RNAs within EVs and the heterogeneous and combinatorial nature of EV cargo, it is less possible that a single RNA species acts independently; rather, a subset of EV‐associated RNAs, including *Gm614*, *Gm10337*, and *Ccdc200*, may cooperatively fine‐tune signaling pathways governing events after hCG stimulation. hCG‐stimulated EVs are possibly taken up by their target cells, and their contents, including these RNAs, may cause the effect. While the specific roles of these transcripts in ovarian cells remain unclear, their accumulation in EVs following hCG stimulation suggests their involvement in hCG signaling networks, including granulosa cells.

Granulosa‐cell proliferation is a central process during follicular development and is primarily driven by FSH through direct activation of intracellular pathways regulating cell cycle progression [13, 16, 67]. In contrast, hCG or LH regulates broader ovarian‐cell functions through both direct and indirect pathways [5, 10]. While these mechanisms explain key aspects of gonadotropin action, they may not fully account for the coordinated cellular responses within the follicular microenvironment. Emerging evidence highlights EVs as important mediators of intercellular communication in the ovary, capable of transferring bioactive cargos, including RNAs, between follicular cells [33, 50, 68]. Our findings support a model in which EVs and their RNA cargos act as intermediaries of hCG signaling, with multiple EV‐associated RNAs functioning cooperatively to fine‐tune signaling pathways within granulosa cells. There may be a possibility that EVs from hCG‐stimulated cells will be applied in some way to the regulation of follicles.

There are several limitations in this study besides the points already mentioned. First, all our data were obtained using hCG, not LH. Because hCG and LH activate LHCGR through quantitatively and qualitatively different signaling pathways [20, 21, 22], our conclusions could be restricted to hCG‐mediated signaling. Second, ovarian EVs from tissue homogenates contained intracellular membranes. This is probably due to too much homogenization of ovaries, and our ovarian samples are therefore more appropriately considered tissue‐derived EV‐enriched preparations. Third, the pooled RNA‐seq design did not permit biological‐replicate‐based statistical analysis or false‐discovery‐rate estimation. This is mainly due to the small amount of long RNAs from EVs, and we consider the identified transcripts as potential DEGs. Future studies will solve these issues to further establish the physiological relevance and molecular contribution of the identified EV RNAs.

In conclusion, this study demonstrates the changes in the EV RNA composition by hCG stimulation and identified potential hCG‐associated EV RNAs. Among them, *Gm614*, one of the EV RNAs, showed the association with increased recipient‐cell number at the examined time points, whereas *Ccdc200* and *Gm10337* showed time‐specific associations. The findings support the notion that hCG stimulation changes the RNA composition within EVs, possibly influencing ovarian intercellular communication with an effect on granulosa‐cell proliferation or survival. The study provides new insight into the regulatory mechanism of follicular development after hCG stimulation.

## Conflict of interest

The authors declare no conflict of interest.

## Author contributions

NP and APK conceived and designed the project. NP, NT, YM, SM, KO, and CF acquired the data. NP, NT, YM, KO, CF, and APK analyzed and interpreted the data. NP and APK wrote the original draft. All authors contributed to the final manuscript.

## Supporting information


**Fig. S1.** Low endogenous *Lhcgr* expression in OV3121 cells.
**Fig. S2.** Schematic overview of EV RNA‐seq workflow and DEG selection criteria.

**Table S1.** Commonly upregulated EV RNA transcripts identified across three experimental models following hCG treatment.
**Table S2.** Commonly downregulated EV RNA transcripts identified across three experimental models following hCG treatment.
**Table S3.** Candidate hCG‐responsive upregulated EV RNA transcripts prioritized based on predefined TPM thresholds.
**Table S4.** Selected protein‐coding EV RNA candidates consistently associated with hCG treatment.

## Data Availability

The raw datasets of RNA‐seq analyses have been deposited to DDBJ Sequence Read Archive under the accession number of BioProject PRJDB42295.
