# Supplementary material for: Potential effect of human chorionic gonadotropin‐induced extracellular vesicle RNAs on mouse ovarian granulosa cells
Source: FEBS Open Bio. 2026 Nov 12:10.1002/2211-5463.70367. Online ahead of print. doi: 10.1002/2211-5463.70367 (PMC13756717; doi:10.1002/2211-5463.70367)
Supplement: Supplementary file 1 [file FEB4-9999-0-s001.pdf]

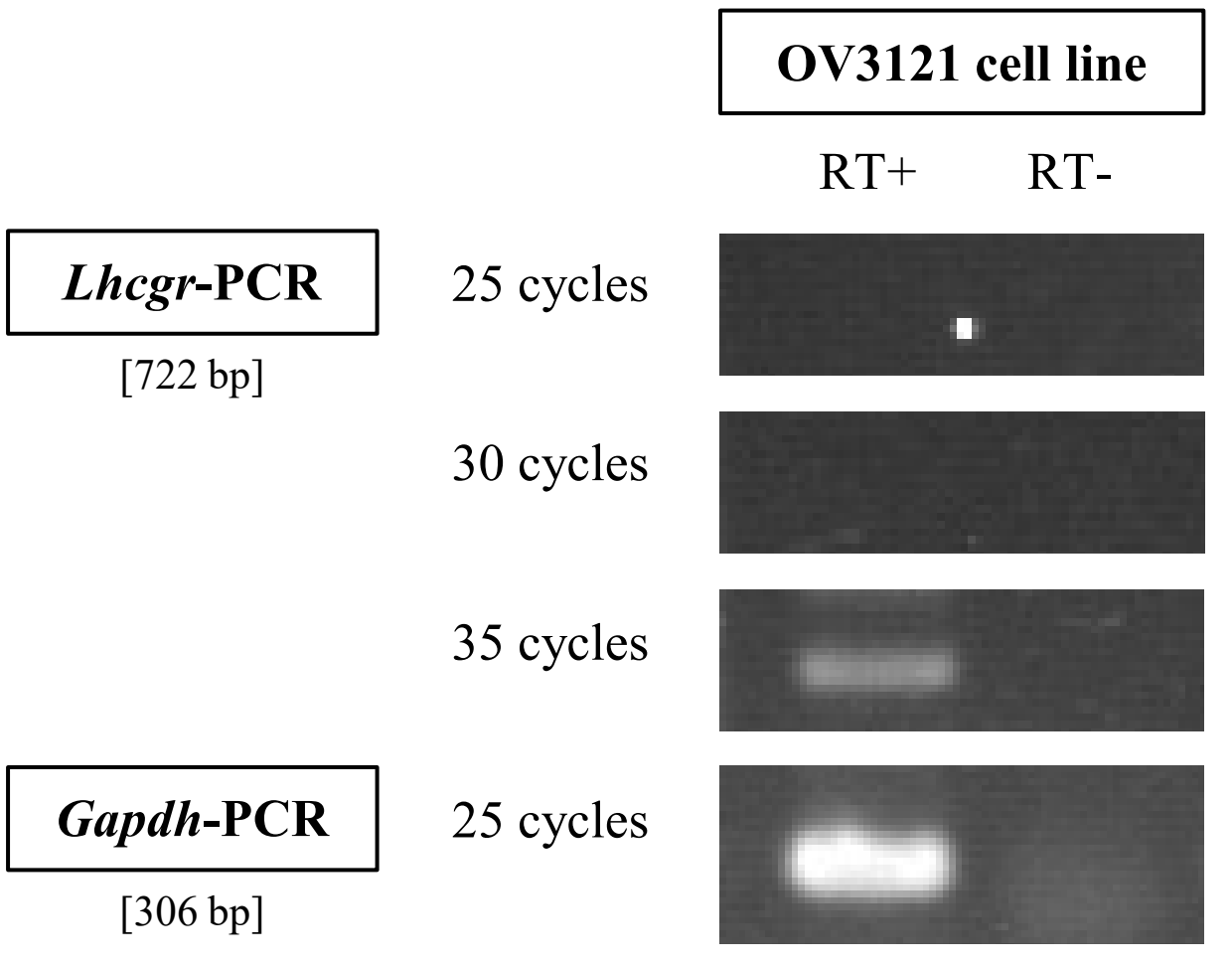

Supplementary Fig. S1. Low endogenous *Lhcgr* expression in OV3121 cells. RT-PCR analysis was performed with cDNA from OV3121 cells for *Lhcgr* and *Gapdh* expression. PCR products were analyzed on 0.7% agarose gels and visualized by ethidium bromide staining. The cycle numbers are indicated. Endogenous *Lhcgr* expression was low or undetectable across all conditions, which is consistent with the limited responsiveness to hCG in OV3121 cells.

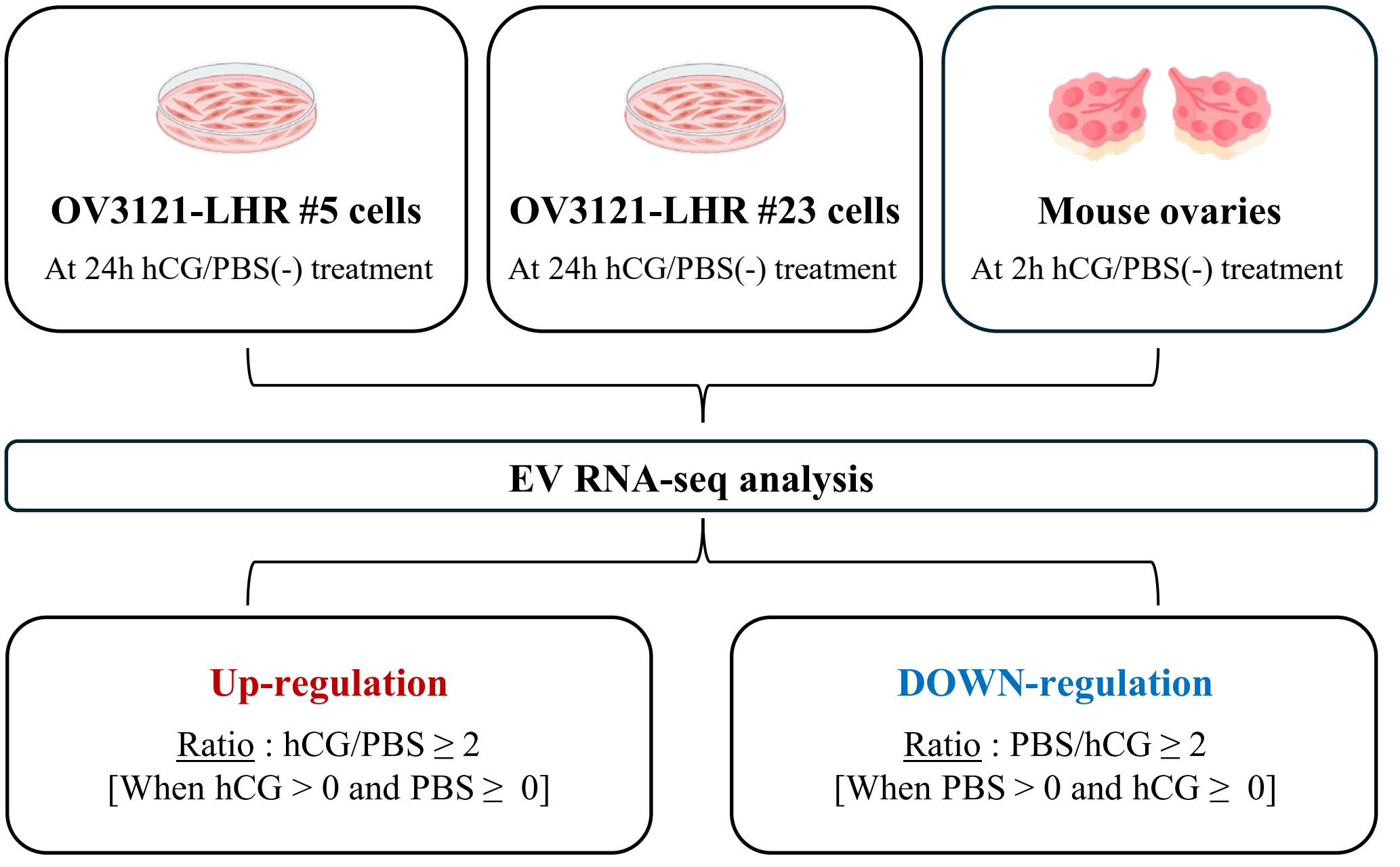

Supplementary Fig. S2. Schematic overview of EV RNA-seq workflow and DEG selection criteria. EVs were isolated from OV3121-LHR #5 cells and OV3121-LHR #23 cells treated with hCG (1 IU/mL) for 24 h, with PBS-treated cells as controls. In parallel, mice were primed with serotropin for 48 h followed by hCG injection, and ovaries were collected 2 h post-hCG. EV RNA was extracted, DNase-treated, quality-controlled, and subjected to RNA-seq analysis. Differential expression analysis was performed based on TPM expression ratios between hCG- and PBS-treated groups. Transcripts showing ≥2-fold upregulation (hCG/PBS) or ≥2-fold downregulation (PBS/hCG) were defined as potential DEGs. Icons used in the illustrations were obtained from BioRender.
